# Genetic evolution and molecular characteristics of avian influenza viruses in Jining from 2018 to 2023

**DOI:** 10.3389/fmicb.2025.1551617

**Published:** 2025-03-27

**Authors:** Mingsheng Zhao, Huixin Dou, Yajuan Jiang, Yongjian Jia, Ying Yue, Libo Li, Shiqing Huang, Meidi Si, Jingjing Wang, Boyan Jiao, Xiaoyu Wang

**Affiliations:** ^1^Institute of Immunology and Molecular Medicine, Jining Medical University, Jining, China; ^2^Department of Microbiological Laboratory, Jining Center for Disease Control and Prevention, Jining, China; ^3^Department of Infectious Disease Control, Jining Center for Disease Control and Prevention, Jining, China

**Keywords:** avian influenza virus, H5N1, H5N6, H7N9, H9N2, genetic evolution, molecular characteristics

## Abstract

**Objective:**

This study aimed to analyze the genetic evolution and molecular characteristics of H5, H7, and H9 subtypes of avian influenza viruses in the external environment of poultry in Jining from 2018 to 2023, providing scientific evidence for the prevention and control of avian influenza.

**Methods:**

Positive samples of H5, H7, and H9 subtypes, collected from the poultry external environment in Jining between 2018 and 2023, were subjected to real-time quantitative PCR. Samples with cycle threshold (CT) values below 30 were selected for influenza virus capture and whole-genome sequencing. Phylogenetic analysis was conducted using bioinformatics software to construct an evolutionary tree, and amino acid mutation sites in the avian influenza virus sequences were analyzed.

**Results:**

Whole-genome sequencing was completed for seven H5N1 strains, four H5N6 strains, one H7N9 strain, and 30 H9N2 strains. Homology analysis revealed that the nucleotide and amino acid sequences of the H5N1 subtype exhibited lower homology with those of the H5N6, H7N9, and H9N2 subtypes, indicating a greater genetic distance. Phylogenetic and molecular characteristic analyses showed that the seven H5N1 strains, four H5N6 strains, and one H7N9 strain were highly pathogenic, while all 30 H9N2 strains were low pathogenic. No mutations were identified at most receptor-binding sites, such as Q226L and G228S, in the H5N1, H5N6, and H7N9 strains, indicating limited mutation at these sites. However, some mutations were observed, suggesting that the virus retained some binding affinity for the human receptor *α*-2,6Gal. In contrast, mutations at receptor-binding sites, including G186V, A190T, and Q226L, were found in most of the H9N2 strains, increasing their likelihood of binding to *α*-2,6Gal and indicating a higher potential for human infection.

**Conclusion:**

The H5, H7, and H9 subtypes of avian influenza viruses are undergoing continuous dynamic evolution and exhibit significant genetic diversity. Enhanced monitoring of viral molecular evolution and research into cross-host transmission are essential.

## 1 Introduction

Avian influenza virus (AIV), a member of the Orthomyxoviridae family under the Influenza A genus, possesses a genome comprising eight gene segments (Lee and Saif, 2009). Based on antigenic differences in the viral glycoproteins hemagglutinin (HA) and neuraminidase (NA), AIV is classified into multiple subtypes (Webby and Uyeki, 2024). To date, 16 HA subtypes (H1–H16) and 9 NA subtypes (N1–N9) have been identified, primarily circulating in wild waterfowl species, especially those in the Anatidae family (e.g., ducks, geese, swans, gulls, and shorebirds) (Uyeki and Peiris, 2019). AIV is further categorized into highly pathogenic (HPAI) and low pathogenic (LPAI) subtypes based on its virulence in poultry (Spackman, 2020). Due to mutations in receptor-binding sites, AIV can cross species barriers, leading to direct human infection. The first human case of avian influenza was reported in Hong Kong in 1997, marking it as a zoonotic disease that poses significant threats to both the poultry industry and public health (Uyeki and Peiris, 2019).

This study aimed to analyze the genetic evolution and molecular characteristics of H5, H7, and H9 AIV subtypes in Jining’s external environment from 2018 to 2023. The findings provide insights into mutation patterns and evolutionary trends of AIVs in the region, contributing vital data for avian influenza prevention and control.

## 2 Materials and methods

### 2.1 Sample collection

Following the “National Surveillance Program for Occupational Exposure Populations and Environmental Monitoring of Highly Pathogenic Avian Influenza (2011 version),” four regions in Jining—Jinxiang County, Wenshang County, Yanzhou District, and Zoucheng City—were designated as monitoring sites. Samples were collected from five environmental categories: urban and rural live poultry markets, large-scale poultry farms, free-range poultry areas, poultry slaughterhouses, and wild bird habitats. Collected samples included feces, cage surface swabs, cutting boards for slaughtered poultry, poultry cleaning wastewater, drinking water for poultry, and other environmental specimens. Samples were collected by trained professional technicians. Ten samples were collected quarterly at each monitoring site. A total of 200 samples were collected in 2018, 158 in 2019, 162 in 2020, 160 in 2021, 160 in 2022, and 200 in 2023, amounting to a total of 1,040 samples over the six-year period.

### 2.2 Nucleic acid detection

Avian influenza environmental samples from Jining City between 2018 and 2023 were collected, and nucleic acids were extracted using automatic nucleic acid extraction system (BioPerFectus). Initial detection was performed using a real-time fluorescent PCR assay kit for influenza A virus nucleic acids (XABT) through reverse transcription quantitative real-time PCR (RT-qPCR). The reaction conditions were as follows: 50°C for 10 min (1 cycle); 95°C for 30 s (1 cycle); 95°C for 5 s, 60°C for 30 s with fluorescence acquisition (45 cycles). Influenza A-positive samples were then screened. Subsequently, RT-qPCR was conducted using a multiplex real-time fluorescent quantitative PCR assay kit for H5, H7, and H9 subtypes of avian influenza virus nucleic acids (XABT) under the same reaction conditions. Samples with a cycle threshold (CT) value less than 30 were selected for whole-genome sequencing of avian influenza virus. Negative and positive controls were included in each assay as part of quality control for nucleic acid detection. The detection limit of the assay kit was 500 copies/mL, with no cross-reactivity with other pathogens, and the coefficient of variation for the detection precision reference material was less than 5%.

### 2.3 Whole-genome sequencing

AIV nucleic acids were captured using the Influenza A Virus Whole Genome Capture Kit (Micro Future) under the following program: 42°C for 60 min (1 cycle); 94°C for 2 min (1 cycle); 94°C for 30 s, 44°C for 30 s, and 68°C for 3 min (5 cycles); 94°C for 30 s, 57°C for 30 s, and 68°C for 3 min (40 cycles); 72°C for 10 min (1 cycle); and cooling to 4°C. Captured products were purified using VAHTS DNA Clean Beads (Vazyme), and nucleic acid quantification was performed using the Qubit 3 Fluorometer (Invitrogen). The cDNA was fragmented using the Nextera XT DNA Library Preparation Kit (Illumina) with the following program: 55°C for 5 min, cooled to 10°C. Adapter ligation was conducted using the Nextera XT Index Kit v2 Set A (Illumina) under the following program: 72°C for 3 min (1 cycle); 95°C for 30 s (1 cycle); 95°C for 10 s, 55°C for 30 s, and 72°C for 30 s (12 cycles); 72°C for 5 min (1 cycle); and cooling to 10°C. Whole-genome sequencing was performed using the Illumina NextSeq2000 Sequencer and the P1 Reagent Kit (300 cycles).

### 2.4 Sequence analysis

Sequencing data were assembled and analyzed using CLC Genomics Workbench 24. Reference strains and candidate vaccine sequences for H5N1, H5N6, H7N9, and H9N2 subtypes were obtained from the GISAID database. Sequence alignment was conducted using MEGA11 and BioEdit software. Neighbor-Joining phylogenetic trees were constructed using MEGA11 software, with parameters set to 1,000 bootstrap replications and the Maximum Composite Likelihood model. Homology analysis of nucleotide and amino acid sequences between samples and vaccine strains was performed using MegAlign (DNASTAR) and MEGA11. Key amino acid mutations were identified using MEGA11 and BioEdit software. The data presented in the study are deposited in the e Global Initiative on Sharing All Influenza Data (GISAID)^1^ repository.

### 2.5 Statistical analysis

Statistical analysis of the data was conducted using SPSS 24.0 software. Intergroup comparisons were performed using the χ^2^ test, with *p* < 0.05 considered as statistically significant differences.

## 3 Results

### 3.1 Surveillance of avian influenza virus

From 2018 to 2023, a total of 1,040 environmental samples were tested for avian influenza in Jining City, with 160 positive samples for influenza A virus, resulting in a positivity rate of 15.38%. Among these, 16 samples were identified as H5 subtype, with a positivity rate of 1.54%; 10 samples as H7 subtype, with a positivity rate of 0.96%; 105 samples as H9 subtype, with a positivity rate of 10.10%; 13 samples as H5/H9 mixed positivity, with a rate of 1.25%; 4 samples as H7/H9 mixed positivity, with a rate of 0.38%; no H5/H7 mixed positivity was detected; 1 sample showed a mixed positivity of H5/H7/H9, with a rate of 0.10%; and 11 samples were classified as untyped A-type, with a rate of 1.06%. The highest number of positive samples was found for the H9 subtype, followed by H5 and H7 subtypes. The differences in positivity rates across subtypes for environmental samples collected from 2018 to 2023 in Jining City were statistically significant (*χ*^2^ = 216.215, *p* < 0.001; Supplementary Table 1).

**Figure 1:**
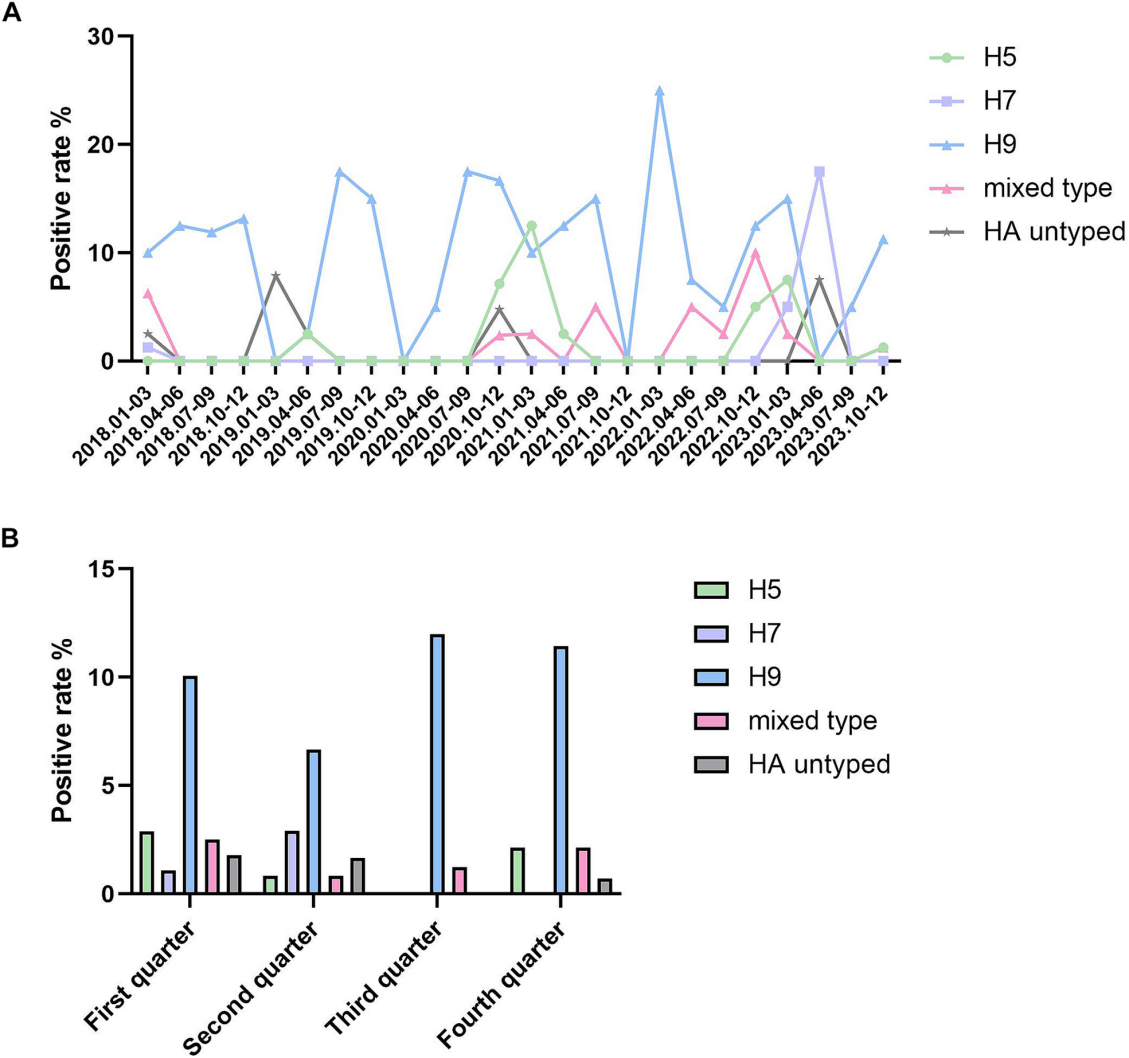
Positive rate of avian influenza viruses in Jining from 2018 to 2023. **(A)** The positive rate of different HA subtypes of avian influenza viruses in Jining from 2018 to 2023. The *X*-axis represents the positive rate, and each point on the *Y*-axis corresponds to each quarter of every year from 2018 to 2023. **(B)** The quarterly positive rate of different HA subtypes of avian influenza viruses in Jining from 2018 to 2023. The *X*-axis represents the positive rate, while the *Y*-axis groups all data from 2018 to 2023 into four quarters. Statistical analysis was performed using SPSS 24.0 software. Intergroup comparisons were conducted using the χ^2^ test, with *p* < 0.05 indicating statistically significant differences.

The positivity rates of various subtypes of avian influenza virus in different years are summarized in Supplementary Table 1 and Figure 1A. The H9 subtype was detected each year, with a relatively stable positivity rate; the H7 subtype was only detected in 2018 and 2023; while the H5 subtype was not detected in 2018, it was present in all subsequent years from 2019 to 2022. Mixed and untyped A-type positives were also observed. Among mixed-positive cases, H5/H9 mixed positivity was more common, with detections in all years except 2019. A mixed positivity of H5/H7/H9 was observed in one sample in 2018. No H5/H7 mixed positivity was detected. The differences in the positivity rates of different subtypes across the 6 years were statistically significant (*χ*^2^ = 63.509, *p* < 0.001).

Surveillance of avian influenza virus subtypes across different quarters is presented in Supplementary Table 1 and Figure 1B. In the first and second quarters, all subtypes of the avian influenza virus were detected. In the third quarter, only the H9 subtype and mixed positives were found, with the mixed positives including two H5/H9 and one H7/H9 cases. In the fourth quarter, the H7 subtype was not detected, but the mixed positives included four H5/H9 and two H7/H9 cases. The H9 subtype of avian influenza virus was the most frequently detected across all four quarters. Positive detections of the H9 subtype were most common in the third quarter, while the H7 subtype exhibited a peak in the second quarter. In contrast, the H5 subtype was most frequently detected in the first quarter. The differences in positivity rates across the four quarters were statistically significant (*χ*^2^ = 35.657, *p* < 0.01).

### 3.2 Homology analysis of avian influenza viruses

For the seven H5N1 strains, the highest nucleotide homology was observed in the NA gene segment (91.60–100%), with the smallest average genetic distance of 0.028. Among the eight gene segments, only the NS gene exhibited lower amino acid homology compared to nucleotide homology. Compared with the A/Cambodia/X0810301/2013 vaccine strain, the PA gene segment had the highest nucleotide homology (89.51–94.98%) with the smallest average genetic distance of 0.079, while the MP gene segment exhibited the highest amino acid homology (94.70–99.20%) (Table 1).

**Table 1:** Homology analysis of H5N1 and H5N6 strains.

Type	H5N1						H5N6					
Gene	Sequence comparison			Compared to A/Cambodia/X0810301/2013 vaccine strain			Sequence comparison			Compared to A/Guangdong/18SF020/2018 vaccine strain		
	Nucleotide homology	Average genetic distance	Amino acid homology	Nucleotide homology	Average genetic distance	Amino acid homology	Nucleotide homology	Average genetic distance	Amino acid homology	Nucleotide homology	Average genetic distance	Amino acid homology
HA	86.63–100%	0.048	90.57–100%	86.50–88.64%	0.127	90.57–91.15%	95.93–98.58%	0.027	95.68–98.58%	97.18–98.70%	0.021	97.86–98.58%
NA	91.60–100%	0.028	93.22–100%	91.16–94.15%	0.080	90.18–91.19%	94.94–99.13%	0.031	95.52–99.78%	94.47–96.28%	0.046	95.08–95.99%
MP	89.98–100%	0.058	95.54–100%	90.30–97.50%	0.082	94.70–99.20%	98.56–99.80%	0.009	99.60–100%	98.56–99.60%	0.009	99.20–99.60%
NS	91.38–100%	0.043	86.24–100%	90.98–94.42%	0.081	87.37–93.44%	97.54–98.78%	0.020	97.19–100%	98.17–98.66%	0.0150	97.19–98.81%
NP	90.33–100%	0.042	98.18–100%	89.86–95.57%	0.088	95.57–98.79%	97.55–99.87%	0.015	99.16–100%	98.04–99.20%	0.013	98.99–99.60%
PA	87.92%-97.28	0.091	96.29–100%	89.51–94.98%	0.079	96.15–98.24%	90.88–100%	0.043	94.89–98.45%	89.51–93.43%	0.089	96.15–99.02%
PB1	86.53–100%	0.074	96.77–100%	87.51–94.61%	0.111	97.62–98.47%	92.39–97.29%	0.050	97.26–99.20%	92.47–97.71%	0.039	97.26–99.34%
PB2	90.11–100%	0.045	97.71–100%	82.64–83.67%	0.167	96.83–97.21%	93.44–98.36%	0.040	97.30–99.34%	93.78–98.45%	0.029	97.44–99.34%

Among the four H5N6 strains, the MP gene segment showed the highest nucleotide homology (98.56–99.80%) with the smallest average genetic distance of 0.009. Compared to the A/Guangdong/18SF020/2018 vaccine strain, the MP gene segment exhibited nucleotide homology of 98.56–99.60%, with the smallest genetic distance of 0.009. The MP gene segment also had the highest amino acid homology (99.20–99.60%) (Table 1).

For the single H7N9 strain, the PB2 gene segment exhibited the highest nucleotide homology (97.49%) compared to the A/Gansu/23277/2019 vaccine strain, with the smallest average genetic distance of 0.025. The PB2 gene segment had the highest amino acid homology (98.53%) (Table 2).

**Table 2:** Homology analysis of H7N9 and H9N2 strains.

Type	H7N9			H9N2					
Gene	Compared to A/Gansu/23277/2019 vaccine strain			Sequence comparison			Compared to A/Oman/2747/2019 vaccine strain		
	Nucleotide homology	Average genetic distance	Amino acid homology	Nucleotide homology	Average genetic distance	Amino acid homology	Nucleotide homology	Average genetic distance	Amino acid homology
HA	96.02%	0.040	96.21%	90.15–100%	0.055	91.04–100%	75.72–80.52%	0.220	83.51–87.63%
NA	97.22%	0.028	95.60%	93.33–100%	0.037	93.68–100%	77.18–83.15%	0.203	81.42–87.72%
MP	97.28%	0.027	98.00%	92.51–100%	0.030	94.70–100%	88.17–90.21%	0.110	93.86–94.70%
NS	97.20%	0.028	95.24%	93.19–100%	0.024	91.09–100%	83.57–88.14%	0.150	75.53–81.30%
NP	97.40%	0.026	98.38%	93.19–100%	0.033	97.56–100%	86.25–89.21%	0.127	95.69–97.15%
PA	96.89%	0.031	98.31%	89.56–100%	0.050	94.77–100%	83.76–89.60%	0.134	94.69–96.57%
PB1	97.02%	0.030	98.40%	87.70–100%	0.065	95.95–100%	84.84–87.14%	0.140	95.31–97.33%
PB2	97.49%	0.025	98.53%	89.28–99.96%	0.058	94.91–100%	83.72–86.13%	0.153	95.29–97.59%

Among the 30 H9N2 strains, the NS gene segment displayed the highest nucleotide homology (93.19–100%) with the smallest average genetic distance of 0.024. For the eight gene segments, only the NS gene had lower amino acid homology compared to nucleotide homology, while the remaining segments showed higher amino acid homology. Compared with the A/Oman/2747/2019 vaccine strain, the MP gene segment had the highest nucleotide homology (88.17–90.21%) with the smallest genetic distance of 0.110. The PB2 gene segment exhibited the highest amino acid homology (95.29–97.59%) (Table 2).

### 3.3 Genetic evolution analysis

#### 3.3.1 HA gene evolution analysis

##### 3.3.1.1 H5 subtype HA gene evolution analysis

**Figure 2:**
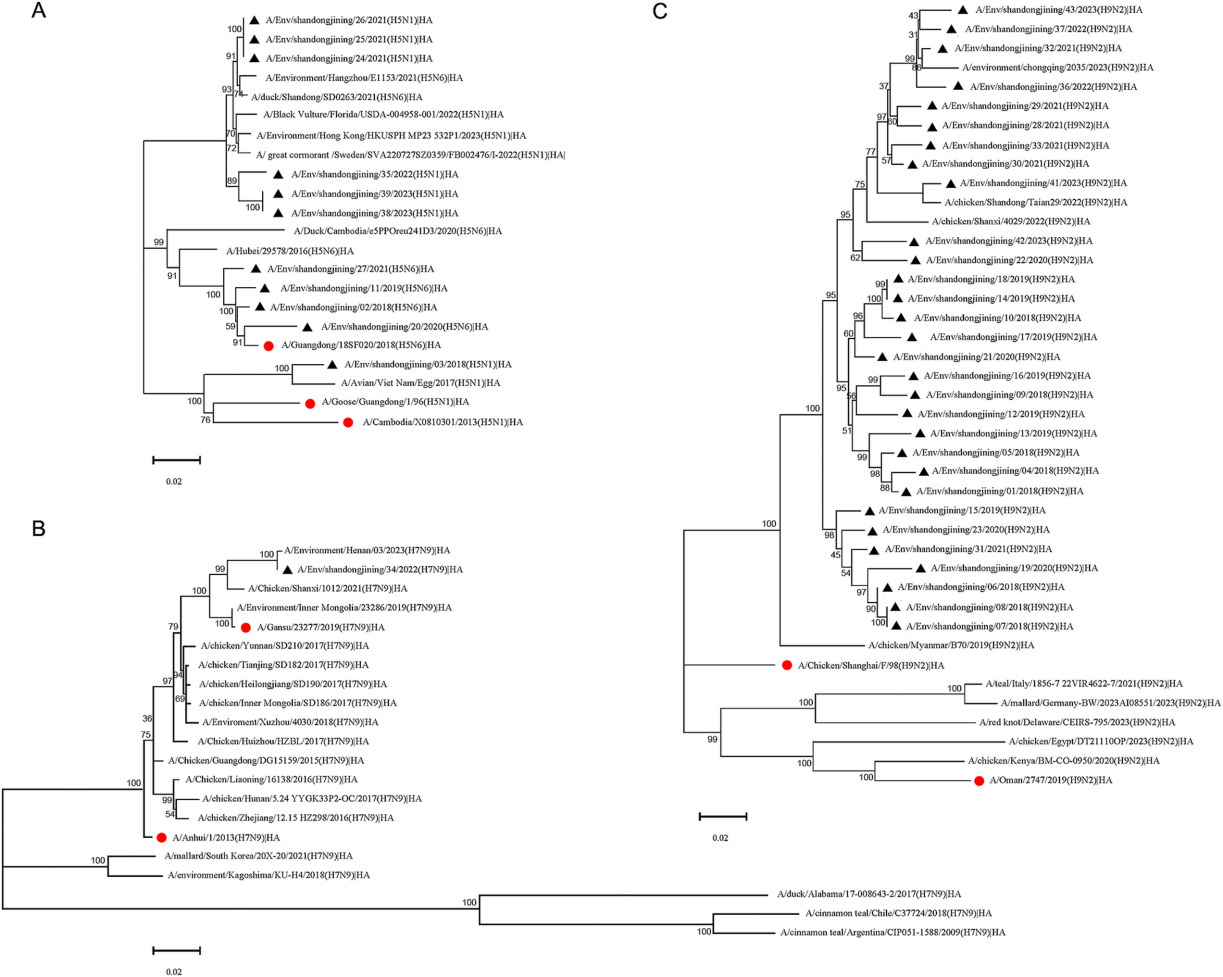
Phylogenetic tree of HA gene of H5, H7N9 and H9N2 subtype avian influenza virus. Phylogenetic analysis of the HA nucleotide sequences of H5, H7N9 and H9N2 avian influenza viruses in Jining during 2018–2023. “●” represents the vaccine strain. “▲” represents strains sequenced in this study. **(A)** H5 subtype HA phylogenetic tree. **(B)** H7N9 subtype HA phylogenetic tree. **(C)** H9N2 subtype HA phylogenetic tree.

Eleven H5 subtype avian influenza virus strains were sequenced, including seven H5N1 and four H5N6 strains. Phylogenetic analysis based on HA gene sequences showed that the four H5N6 strains belonged to the 2.3.4.4 h evolutionary branch (Cho et al., 2024), alongside the H5N6 vaccine strain A/Guangdong/18SF020/2018, while the A/Hubei/29578/2016 strain clustered in a separate 2.3.4.4d branch (Bi et al., 2016). Of the seven H5N1 strains, six were grouped with H5N6 reference strains from Hangzhou and Shandong, as well as H5N1 reference strains from Florida, Hong Kong, and Sweden, all within the 2.3.4.4b branch (Sun et al., 2018; Yang et al., 2023; Figure 2A). The remaining H5N1 strain clustered with H5N1 vaccine strains A/Goose/Guangdong/1/96, A/Cambodia/X0810301/2013, and the Vietnamese reference strain but showed no subtype differentiation (Figure 2A).

##### 3.3.1.2 H7N9 subtype HA gene evolution analysis

A single H7N9 strain was sequenced and analyzed using HA gene sequences from domestic and international H7N9 vaccine and reference strains. The sequenced strain clustered with vaccine strains A/Anhui/1/2013, A/Gansu/23277/2019, and domestic reference strains. Reference strains from South Korea and Japan formed a distinct cluster, while those from the United States, Chile, and Argentina grouped in separate branches (Figure 2B).

##### 3.3.1.3 H9N2 subtype HA gene evolution analysis

Thirty H9N2 subtype avian influenza virus strains were sequenced. Phylogenetic analysis showed that these strains clustered with H9N2 reference strains from Chongqing, Shandong, and Shaanxi, China, while reference strains from Europe, North America, Africa, and the Middle East formed separate branches, indicating a more distant genetic relationship to Asian strains (Figure 2C).

#### 3.3.2 NA gene evolution analysis

##### 3.3.2.1 H5N1 subtype NA gene evolution analysis

**Figure 3:**
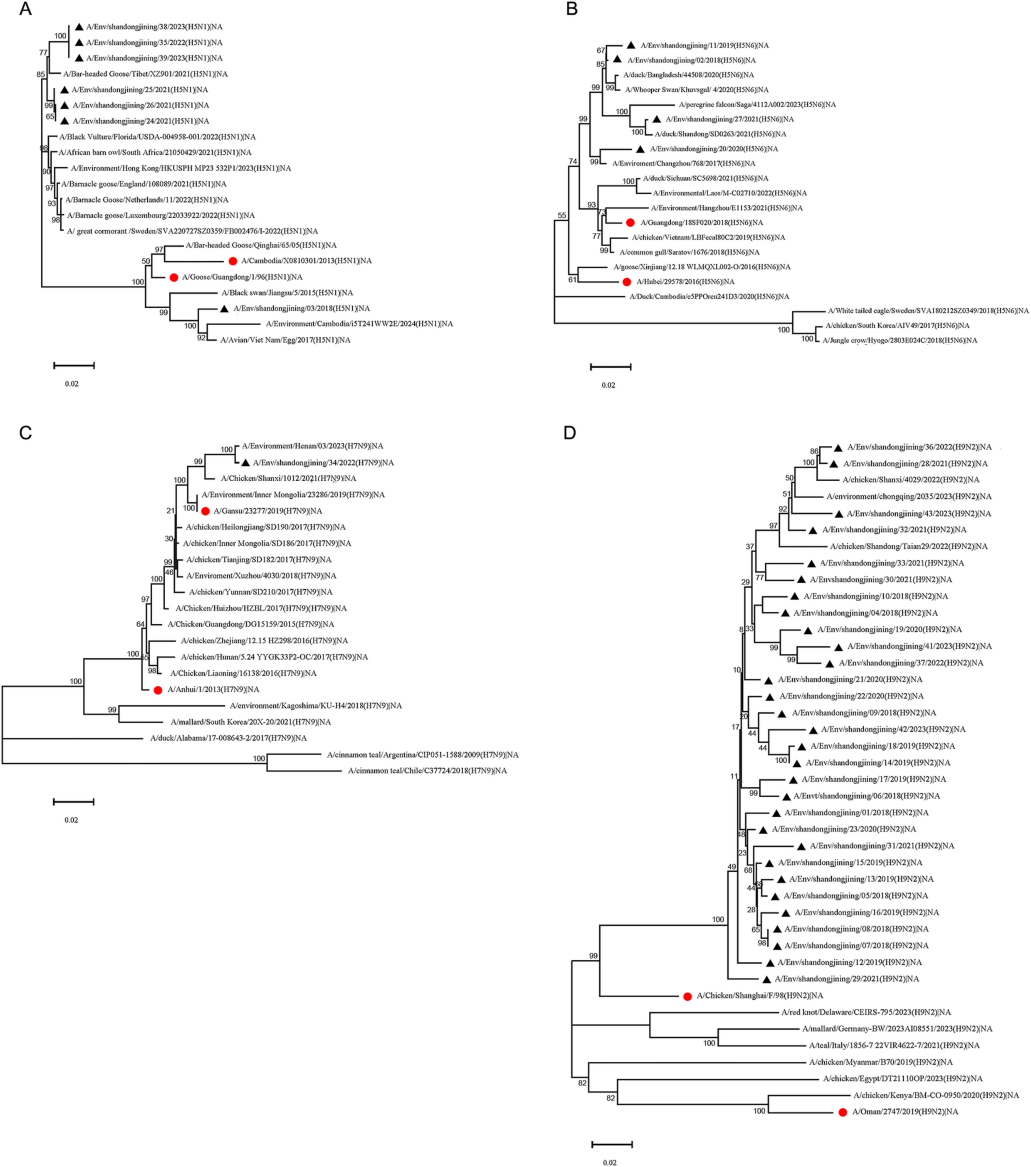
Phylogenetic tree of NA gene of H5N1, H5N6, H7N9 and H9N2 subtype avian influenza virus. Phylogenetic analysis of the NA nucleotide sequences of H5N1, H5N6, H7N9 and H9N2 avian influenza viruses in Jining during 2018–2023. “●” represents the vaccine strain. “▲” represents strains sequenced in this study. **(A)** H5N1 subtype NA phylogenetic tree. **(B)** H5N6 subtype NA phylogenetic tree. **(C)** H7N9 subtype NA phylogenetic tree. **(D)** H9N2 subtype NA phylogenetic tree.

Seven H5N1 subtype strains were analyzed for NA gene evolution. The A/Env/shandongjining/03/2018 strain clustered with vaccine strains A/Cambodia/X0810301/2013 and A/Goose/Guangdong/1/96, while the remaining six strains grouped with reference strains from Europe, Africa, and Asia (Figure 3A).

##### 3.3.2.2 H5N6 subtype NA gene evolution analysis

Phylogenetic analysis of the NA gene in four H5N6 strains showed clustering with vaccine and reference strains from multiple Asian countries. Reference strains from Sweden, South Korea, and Japan (Hyogo Prefecture) formed distinct branches (Figure 3B).

##### 3.3.2.3 H7N9 subtype NA gene evolution analysis

The single H7N9 strain sequenced in this study clustered with vaccine strains A/Anhui/1/2013, A/Gansu/23277/2019, and domestic reference strains. Reference strains from South Korea and Japan formed one cluster, while strains from the United States formed another. Strains from Chile and Argentina clustered in a distinct branch (Figure 3C).

##### 3.3.2.4 H9N2 subtype NA gene evolution analysis

Phylogenetic analysis of the NA gene in 30 H9N2 strains revealed clustering with reference strains from Chongqing, Shandong, and Shaanxi, China. The vaccine strain A/Chicken/shanghai/F/98 formed a separate branch. Reference strains from Europe and North America clustered together, while those from Africa and the Middle East formed a distinct branch (Figure 3D).

#### 3.3.3 PA, MP, NP, NS, PB1 and PB2 gene evolution analysis for H5N1, H5N6, H7N9, and H9N2 subtypes

Phylogenetic trees were constructed for the PA, MP, NP, NS, PB1, and PB2 gene sequences of 7 H5N1, 4 H5N6, 1 H7N9, and 30 H9N2 avian influenza virus strains, as shown in Supplementary Figures 1–6.

The phylogenetic tree of the PA gene revealed that the 42 strains sequenced in this study clustered within a major evolutionary branch alongside strains from Asian countries such as Myanmar and Mongolia, as well as Oman, Germany, and Florida, USA, indicating close genetic relationships. In contrast, strains from Argentina and Delaware, USA, formed a separate evolutionary branch (Supplementary Figure 1).

The MP gene phylogenetic tree showed three main branches. Among the strains sequenced in this study, 30 clustered with strains from several Chinese cities and Myanmar within one branch. The vaccine strains A/Oman/2747/2019 and A/Chicken/Shanghai/F/98 formed a separate branch, while the remaining 12 strains clustered with strains from Vietnam, Cambodia, other Asian countries, as well as from the Americas and Germany (Supplementary Figure 2).

The NP gene phylogenetic tree demonstrated that the 42 strains sequenced in this study were in the same branch as strains from Asian, Middle Eastern, and European countries, whereas strains from the Americas constituted a distinct branch (Supplementary Figure 3).

The NS gene phylogenetic tree indicated that the 42 strains sequenced in this study clustered with strains from Myanmar, Mongolia, and other Asian countries, as well as Oman and Florida, USA, suggesting close genetic relationships. Strains from Argentina and Delaware, USA, were located in the same evolutionary branch, while strains from Germany and the vaccine strain A/Goose/Guangdong/1/96 formed a separate divergent branch (Supplementary Figure 4).

The PB1 gene phylogenetic tree showed that the 42 strains sequenced in this study were closely related to strains from Asian, Middle Eastern, European, and American countries, all belonging to a large, shared evolutionary branch (Supplementary Figure 5).

The PB2 gene phylogenetic tree indicated that the 42 strains sequenced in this study were in the same major evolutionary branch as strains from Asian, European, and Middle Eastern countries, with close genetic relationships. In contrast, strains from the Americas formed a distinct evolutionary branch (Supplementary Figure 6).

### 3.4 Protein molecular characterization analysis

#### 3.4.1 HA protein molecular characterization analysis

Analysis of HA protein cleavage sites in seven H5N1 and four H5N6 strains revealed the following patterns: seven strains had LRERRRKR\GLF, one had QRERRRKR\GLF, and three had LREKRRKR\GLF (Zhang et al., 2009). These cleavage sites contain consecutive basic amino acids characteristic of highly pathogenic avian influenza viruses (Suttie et al., 2019). Similarly, the HA cleavage site of the H7N9 strain was PKRKRAAR\GLF, indicating high pathogenicity (Gu et al., 2017). Conversely, all 30 H9N2 strains had NVPSRSSR\GLF, with only one basic amino acid, indicating low pathogenicity (Dortmans et al., 2013; Table 3).

**Table 3:** Key amino acid positions in HA protein of H5, H7N9 and H9N2 subtype avian influenza viruses.

Type	Virus strain	Cleavage site	Receptor binding site						
			137	160	226	227	228	239	255
H5N1	A/Env/shandongjining/03/2018	QRERRRKR\GLF	A	A	Q	S	G	P	E
	A/Env/shandongjining/24/2021	LREKRRKR\GLF	A	A	Q	R	G	P	E
	A/Env/shandongjining/25/2021	LREKRRKR\GLF	A	A	Q	R	G	P	E
	A/Env/shandongjining/26/2021	LREKRRKR\GLF	A	A	Q	R	G	P	E
	A/Env/shandongjining/35/2022	LRERRRKR\GLF	A	A	Q	R	G	P	E
	A/Env/shandongjining/38/2023	LRERRRKR\GLF	A	A	Q	R	G	P	E
	A/Env/shandongjining/39/2023	LRERRRKR\GLF	A	A	Q	R	G	P	E
H5N6	A/Env/shandongjining/02/2018	LRERRRKR\GLF	A	A	Q	R	G	P	E
	A/Env/shandongjining/11/2019	LRERRRKR\GLF	A	A	Q	R	G	P	E
	A/Env/shandongjining/20/2020	LRERRRKR\GLF	A	A	Q	R	G	P	E
	A/Env/shandongjining/27/2021	LRERRRKR\GLF	A	A	Q	G	G	P	E
H7N9			**160**	**186**	**221**	**224**	**226**	**228**	
	A/Env/shandongjining/34/2022	PKRKRAAR\GLF	T	I	P	N	Q	G	
H9N2			**155**	**183**	**186**		**190**	**226**	
	A/Env/shandongjining/01/2018	NVPSRSSR\GLF	T	N	P		T	L	
	A/Env/shandongjining/04/2018	NVPSRSSR\GLF	T	N	P		T	L	
	A/Env/shandongjining/05/2018	NVPSRSSR\GLF	T	N	P		T	L	
	A/Env/shandongjining/06/2018	NVPSRSSR\GLF	T	X	X		X	L	
	A/Env/shandongjining/07/2018	NVPSRSSR\GLF	T	N	P		A	L	
	A/Env/shandongjining/08/2018	NVPSRSSR\GLF	T	N	P		A	L	
	A/Env/shandongjining/09/2018	NVPSRSSR\GLF	T	N	P		T	L	
	A/Env/shandongjining/10/2018	NVPSRSSR\GLF	T	N	P		T	L	
	A/Env/shandongjining/12/2019	NVPSRSSR\GLF	N	N	P		T	L	
	A/Env/shandongjining/13/2019	NVPSRSSR\GLF	T	N	P		T	L	
	A/Env/shandongjining/14/2019	NVPSRSSR\GLF	T	N	P		T	L	
	A/Env/shandongjining/15/2019	NVPSRSSR\GLF	T	N	P		T	L	
	A/Env/shandongjining/16/2019	NVPSRSSR\GLF	T	N	P		T	L	
	A/Env/shandongjining/17/2019	NVPSRSSR\GLF	T	N	P		T	L	
	A/Env/shandongjining/18/2019	NVPSRSSR\GLF	T	N	P		T	L	
	A/Env/shandongjining/19/2020	NVPSRSSR\GLF	I	N	P		V	L	
	A/Env/shandongjining/21/2020	NVPSRSSR\GLF	T	N	P		T	L	
	A/Env/shandongjining/22/2020	NVPSRSSR\GLF	N	N	P		X	L	
	A/Env/shandongjining/23/2020	NVPSRSSR\GLF	T	N	P		V	L	
	A/Env/shandongjining/28/2021	NVPSRSSR\GLF	N	N	P		T	L	
	A/Env/shandongjining/29/2021	NVPSRSSR\GLF	N	N	P		X	L	
	A/Env/shandongjining/30/2021	NVPSRSSR\GLF	N	N	P		X	L	
	A/Env/shandongjining/31/2021	NVPSRSSR\GLF	T	X	X		X	L	
	A/Env/shandongjining/32/2021	NVPSRSSR\GLF	N	N	P		V	L	
	A/Env/shandongjining/33/2021	NVPSRSSR\GLF	N	N	P		T	L	
	A/Env/shandongjining/36/2022	NVPSRSSR\GLF	N	N	P		V	L	
	A/Env/shandongjining/37/2022	NVPSRSSR\GLF	N	N	P		V	L	
	A/Env/shandongjining/41/2023	NVPSRSSR\GLF	N	N	P		V	L	
	A/Env/shandongjining/42/2023	NVPSRSSR\GLF	X	N	P		T	X	
	A/Env/shandongjining/43/2023	NVPSRSSR\GLF	N	N	P		V	L	

Mutations are marked in red, while positions with undetectable amino acids during sequencing are indicated by a blue “X”. The bold values represent amino acid positions.

Mutations in HA receptor-binding sites influence cross-species transmission. For H5 subtypes, mutations at positions such as S137A, T160S, Q226L, S/R227N, G228S, P239S, and E255K enhance human receptor affinity (SA*α*-2,6Gal) (Russell et al., 2012). The 11 H5 strains showed no mutations at T160, Q226, G228, P239, or E255, preserving avian receptor affinity (SAα-2,3Gal) (Zhang et al., 2013). However, all strains exhibited S137A mutations, and one strain, A/Env/shandongjining/27/2021 (H5N6), showed S/R227G, indicating some human receptor-binding potential (Plaza et al., 2024; Table 3).

For the H7N9 strain, mutations at T160A, G186V, T221P, N224K, Q226L, and G228S are critical for human receptor binding (*α*-2,6Gal) (Dortmans et al., 2013). The sequenced strain exhibited a T221P mutation, enhancing human receptor binding, along with a novel I186 mutation suggesting dual receptor-binding properties (de Graaf and Fouchier, 2014; Yang et al., 2017). Additionally, a novel mutation at 186I (Isoleucine) suggests that this H7N9 strain has dual receptor-binding properties and preferentially binds to the human receptor α-2,6Gal (Guo et al., 2020; Xu et al., 2019; Table 3).

Analysis of key receptor-binding sites in 30 H9N2 strains revealed mutations at T155N, H183N, G186P, A190T, and Q226L. The A/Env/shandongjining/19/2020 strain displayed an I155 mutation, while other strains either mutated to N or remained unchanged (Li et al., 2017; Pu et al., 2015). All strains showed H183N and Q226L mutations. These findings indicate a preference for human receptors (α-2,6Gal), suggesting significant potential for human infection (Li et al., 2020; Sun et al., 2020; Jiang et al., 2012; Table 3).

#### 3.4.2 Molecular characteristics of NA protein

Amino acid deletions in the stalk region of the neuraminidase (NA) protein lead to structural changes that enhance enzymatic activity, increasing viral virulence and improving adaptability and pathogenicity in hosts. Sequence analysis of the NA proteins in H5N1 strains revealed that A/Env/ShandongJining/03/2018 exhibited a 22-amino-acid deletion at positions 49–70, while the remaining six strains showed no deletions in the stalk region. The NA protein of the H5N1 vaccine strain A/Cambodia/X0810301/2013 displayed a 20-amino-acid deletion at positions 49–68 (Chen et al., 2019). Similarly, the four H5N6 strains analyzed had an 11-amino-acid deletion at positions 59–69, suggesting an association with increased virulence and infectivity in avian and human hosts (Samson et al., 2013). Further analysis indicated no mutations at resistance-associated sites (E119D, H274Y, and R292K) in the seven H5N1 and four H5N6 strains, indicating continued sensitivity to neuraminidase inhibitors such as oseltamivir and zanamivir (He et al., 2018; Pulit-Penaloza et al., 2024).

The H7N9 strain analyzed exhibited an amino acid deletion mutation at positions 69–73 of the NA protein, indicating enhanced virulence (Suttie et al., 2019; Shi et al., 2017). No mutations were observed at resistance-associated positions 119, 152, 274, or 292, suggesting retained sensitivity to antiviral agents such as oseltamivir (Wang et al., 2018; Shi et al., 2013).

Analysis of the NA proteins of 30 H9N2 strains revealed varying degrees of deletions at positions 62–68, involving two, three, or four residues (Wang et al., 2021), indicative of increased pathogenicity. Resistance-associated mutations at E119 and R156 were absent, ensuring NA tetramer stability. Furthermore, no mutations were detected at H274, E276, or R292, indicating continued sensitivity to neuraminidase inhibitors (Sun et al., 2013). However, mutations at hemagglutinin-binding sites, including D368N, D369G/S, S402N/D, and Q432K, were observed. All 30 H9N2 strains exhibited an N356D mutation, a key antigenic site, suggesting potential antigenic drift and vaccine escape (Richard et al., 2008; Guo et al., 2009).

#### 3.4.3 Molecular characterization of PA, MP, NP, NS, PB1, and PB2 proteins

The amino acid sites related to human adaptation in PA, MP, NP, NS, PB1, and PB2 proteins were analyzed. Mutations D55N, L268I, and S409N in the PA protein have been reported to enhance the adaptability of avian influenza viruses to humans (Chen et al., 2006). Among the 42 strains sequenced in this study, none exhibited D55N or L268I mutations in the PA protein. However, 22 strains showed the N mutation at position 409, while the remaining 20 strains did not. No V115I, T121A, or T137A mutations were detected in the M1 protein of any strain, and no G16D, L283P, or F313Y mutations were found in the NP protein. Similarly, no R327K or V336I mutations were observed in the PB1 protein, and no T271A, A588I, or E627K mutations were detected in the PB2 protein. Regarding the NS protein, no I81M or E227R mutations were found in any strain. However, at position 215, only strain A/Env/shandongjining/37/2022 exhibited the T mutation, while all other strains showed no mutations (Finkelstein et al., 2007).

## 4 Discussion

Jining, a major city in Shandong Province, China, has a permanent population of over 8.2 million. It is home to Weishan Lake, the largest freshwater lake in northern China, which serves as a critical node along the East Asian-Australasian Flyway, one of the busiest migratory bird routes globally. Additionally, Jining’s large-scale poultry farming presents an environment where wild migratory birds and domestic poultry coexist.

The H5 subtype avian influenza virus (AIV) Clade 2.3.4.4 is divided into eight evolutionary branches (a-h). Since 2014, most H5 subtype AIV strains in China and Southeast Asia have belonged to branches 2.3.4.4d–h. This study identified four H5N6 strains in the 2.3.4.4 h branch, while six of the seven H5N1 strains were classified under the 2.3.4.4b branch, with one strain untyped (Lee et al., 2016). The 2.3.4.4b branch primarily comprises H5N8 strains (Lee et al., 2017), suggesting that 2.3.4.4b H5N1 strains may have arisen through genetic reassortment with H5N8, warranting enhanced surveillance. Phylogenetic analysis of H5 subtype HA genes indicated that six of the seven H5N1 strains clustered with Eurasian strains in the 2.3.4.4b branch, while one strain grouped with Asian strains. The four H5N6 strains were exclusively located in the 2.3.4.4 h branch. NA gene evolution followed similar patterns, indicating a close relationship between H5 subtype AIVs circulating in China and those in Europe. All seven H5N1 and four H5N6 strains were identified as highly pathogenic, with no mutations at receptor-binding amino acid sites T160, Q226, G228, P239, and E255 of the HA protein, suggesting continued sensitivity to avian receptor *α*-2,3Gal (Suttie et al., 2019). However, all 11 H5 strains exhibited an S137A mutation, with one H5N6 strain (A/Env/ShandongJining/27/2021) displaying an additional S/R227G mutation, indicating partial binding affinity to human receptor α-2,6Gal. NA protein sequence analysis revealed deletions in one H5N1 strain and all four H5N6 strains, signifying enhanced virulence and infectivity (Sun et al., 2016; Herfst et al., 2018). None of the 11 H5 strains showed resistance-associated mutations (E119D, H274Y, or R292K), maintaining sensitivity to neuraminidase inhibitors.

The H7 subtype AIV is less prevalent than the H5 and H9 subtypes. The single H7N9 strain sequenced in this study exhibited HA and NA genes clustering with vaccine and Asian reference strains within the same evolutionary branch. This highly pathogenic strain showed no T160A, N224K, Q226L, or G228S mutations in the HA protein, but a T221P mutation enhanced binding affinity to human receptor *α*-2,6Gal while reducing affinity for avian receptor α-2,3Gal (Shi et al., 2013). A novel I186 mutation suggested dual receptor-binding properties, favoring human receptors. The NA protein displayed deletions at positions 69–73 with no resistance-related mutations (119, 152, 274, or 292), ensuring susceptibility to oseltamivir (Dortmans et al., 2013).

The H9 subtype avian influenza virus is globally distributed and classified into Eurasian and American lineages based on HA genes (Peacock et al., 2018). The strains sequenced in this study belonged to the Eurasian Y280-like (G9-like) branch, with all 30 H9N2 strains identified as low pathogenicity viruses. Sequence analysis revealed mutations at key receptor-binding sites, including T155N, H183N, G186V, A190T, and Q226L, which facilitate binding to α-2,6Gal receptors and potentially increase human infectivity. Amino acid deletions at positions 62–68 in the NA protein were observed to varying degrees, correlating with enhanced pathogenicity. No mutations were identified at H274, E276, or R292, indicating sustained sensitivity to neuraminidase inhibitors (Gu et al., 2017).

Avian influenza viruses continuously undergo mutations and reassortments, leading to increased virulence and pathogenicity (Han et al., 2019; Koutsakos et al., 2019). These viruses exhibit dual receptor-binding properties or a preference for human receptors, resulting in antigenic changes (Shi et al., 2023). The transmission of avian influenza between poultry and wild birds, coupled with frequent interspecies spillover, promotes genomic recombination and variation. This enhances adaptability across species and raises the potential for human-to-human transmission (Du et al., 2017). Avian influenza represents a significant threat to poultry farming and human health, posing a substantial challenge for public health prevention and control efforts (Bi et al., 2019). Jining City, with its large-scale poultry farming, active live poultry markets, high population mobility, and proximity to key migratory bird habitats such as Beihu and Nansi Lake, is at particularly high risk. Strengthening surveillance of avian influenza viruses in external environments and conducting research on viral genomic and protein sequences are essential. These measures will aid in understanding the pathogenicity and host-binding characteristics of the virus and tracking changes in its antigenic properties (Spackman, 2020). For poultry, the focus should be on reducing avian influenza infection rates, preventing large-scale outbreaks among birds, minimizing economic losses in the poultry industry, ensuring food safety, and promoting the sustainable development of poultry farming. For humans, the findings provide critical scientific evidence for developing comprehensive prevention and control strategies for avian influenza infections and enhancing early warning systems for potential influenza pandemics (Uyeki and Peiris, 2019).

## Data Availability

The datasets presented in this study can be found in online repositories. The names of the repository/repositories and accession number(s) can be found in the article/Supplementary material.

## Footnotes

^1^ https://gisaid.org

## Supplementary material

SUPPLEMENTARY FIGURE 1 Phylogenetic tree of PA gene of H5N1, H5N6, H7N9, and H9N2 subtype avian influenza viruses. Phylogenetic analysis of the PA nucleotide sequences of H5N1, H5N6, H7N9 and H9N2 avian influenza viruses in Jining during 2018–2023. “●” represents the vaccine strain. “▲” represents strains sequenced in this study.

SUPPLEMENTARY FIGURE 2 Phylogenetic tree of MP gene of H5N1, H5N6, H7N9, and H9N2 subtype avian influenza viruses. Phylogenetic analysis of the MP nucleotide sequences of H5N1, H5N6, H7N9 and H9N2 avian influenza viruses in Jining during 2018–2023. “●” represents the vaccine strain. “▲” represents strains sequenced in this study.

SUPPLEMENTARY FIGURE 3 Phylogenetic tree of NP gene of H5N1, H5N6, H7N9, and H9N2 subtype avian influenza viruses. Phylogenetic analysis of the NP nucleotide sequences of H5N1, H5N6, H7N9 and H9N2 avian influenza viruses in Jining during 2018–2023. “●” represents the vaccine strain. “▲” represents strains sequenced in this study.

SUPPLEMENTARY FIGURE 4 Phylogenetic tree of NS gene of H5N1, H5N6, H7N9, and H9N2 subtype avian influenza viruses. Phylogenetic analysis of the NS nucleotide sequences of H5N1, H5N6, H7N9 and H9N2 avian influenza viruses in Jining during 2018–2023. “●” represents the vaccine strain. “▲”represents strains sequenced in this study.

SUPPLEMENTARY FIGURE 5 Phylogenetic tree of PB1 gene of H5N1, H5N6, H7N9, and H9N2 subtype avian influenza viruses. Phylogenetic analysis of the PB1 nucleotide sequences of H5N1, H5N6, H7N9 and H9N2 avian influenza viruses in Jining during 2018–2023. “●” represents the vaccine strain. “▲” represents strains sequenced in this study.

SUPPLEMENTARY FIGURE 6 Phylogenetic tree of PB2 gene of H5N1, H5N6, H7N9, and H9N2 subtype avian influenza viruses. Phylogenetic analysis of the PB2 nucleotide sequences of H5N1, H5N6, H7N9 and H9N2 avian influenza viruses in Jining during 2018–2023. “●” represents the vaccine strain. “▲” represents strains sequenced in this study.
