# Supplementary figures and images for: Genetic evolution and molecular characteristics of avian influenza viruses in Jining from 2018 to 2023
Source: Front Microbiol. 2025 Mar 27;16:1551617. doi: 10.3389/fmicb.2025.1551617 (PMC11983498; doi:10.3389/fmicb.2025.1551617)

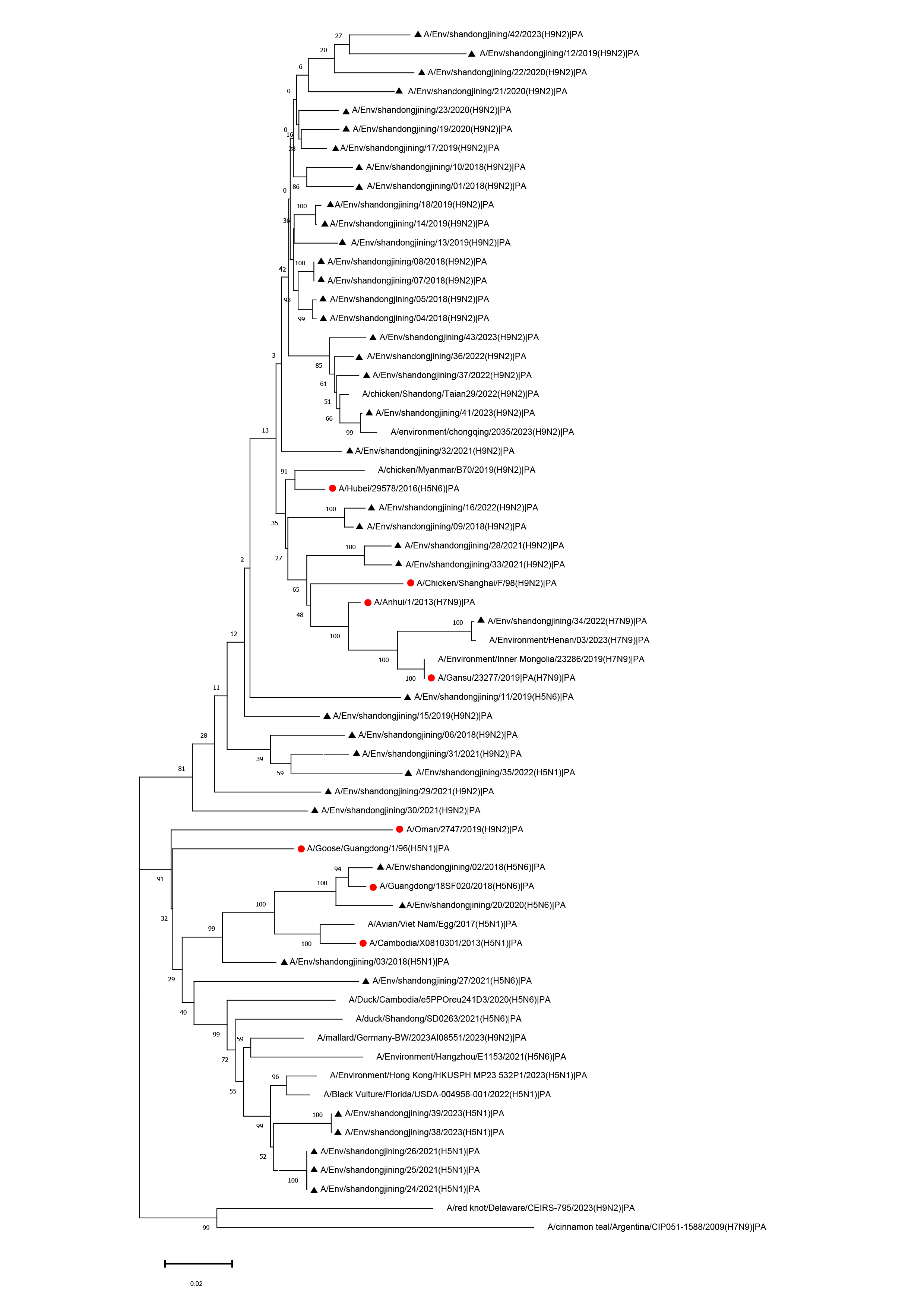

Supplement: SUPPLEMENTARY FIGURE 1 — Phylogenetic tree of PA gene of H5N1, H5N6, H7N9, and H9N2 subtype avian influenza viruses. Phylogenetic analysis of the PA nucleotide sequences of H5N1, H5N6, H7N9 and H9N2 avian influenza viruses in Jining during 2018–2023. “●” represents the vaccine strain. “▲” represents strains sequenced in this study. [file Image_1.TIF]

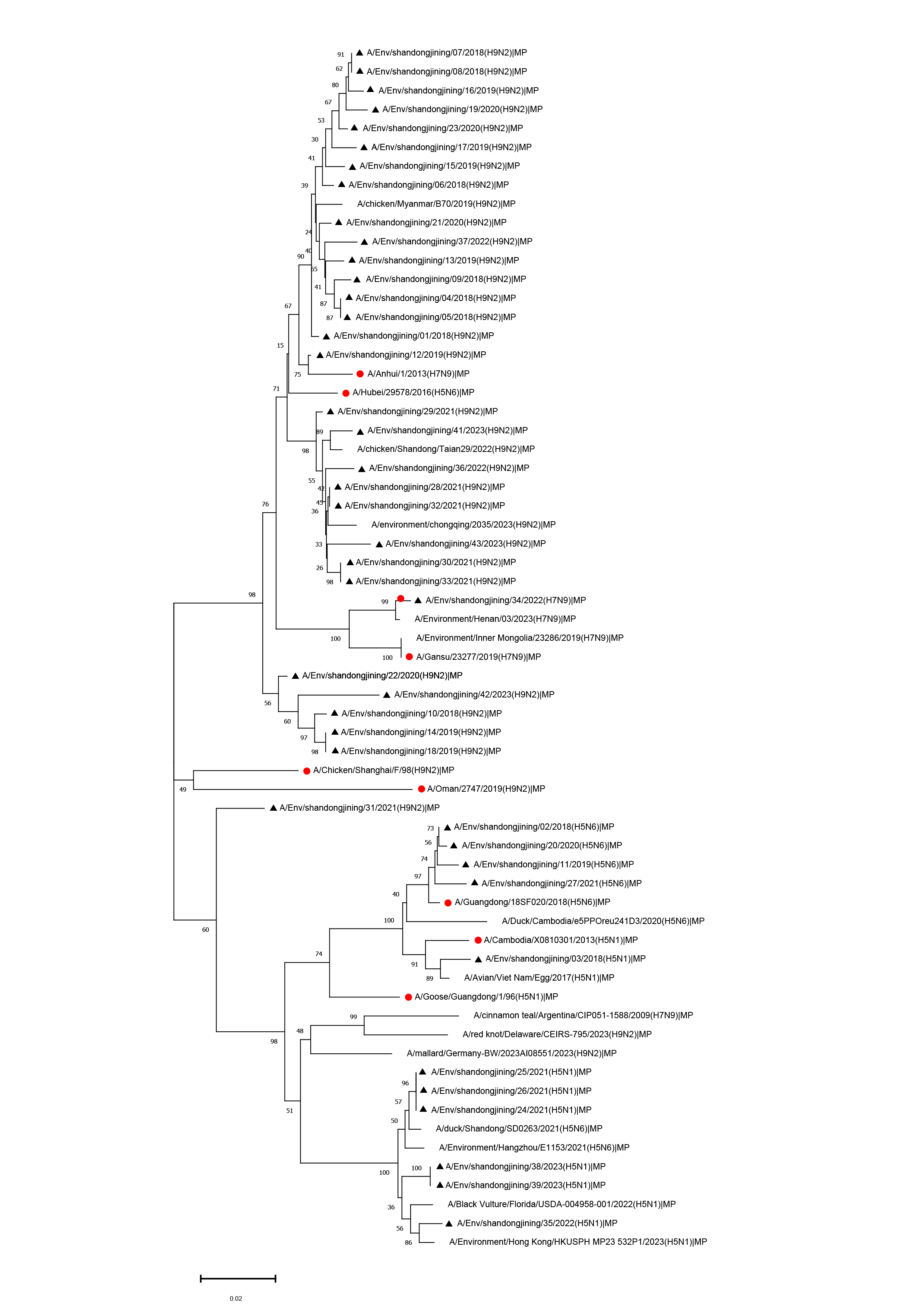

Supplement: SUPPLEMENTARY FIGURE 2 — Phylogenetic tree of MP gene of H5N1, H5N6, H7N9, and H9N2 subtype avian influenza viruses. Phylogenetic analysis of the MP nucleotide sequences of H5N1, H5N6, H7N9 and H9N2 avian influenza viruses in Jining during 2018–2023. “●” represents the vaccine strain. “▲” represents strains sequenced in this study. [file Image_2.TIF]

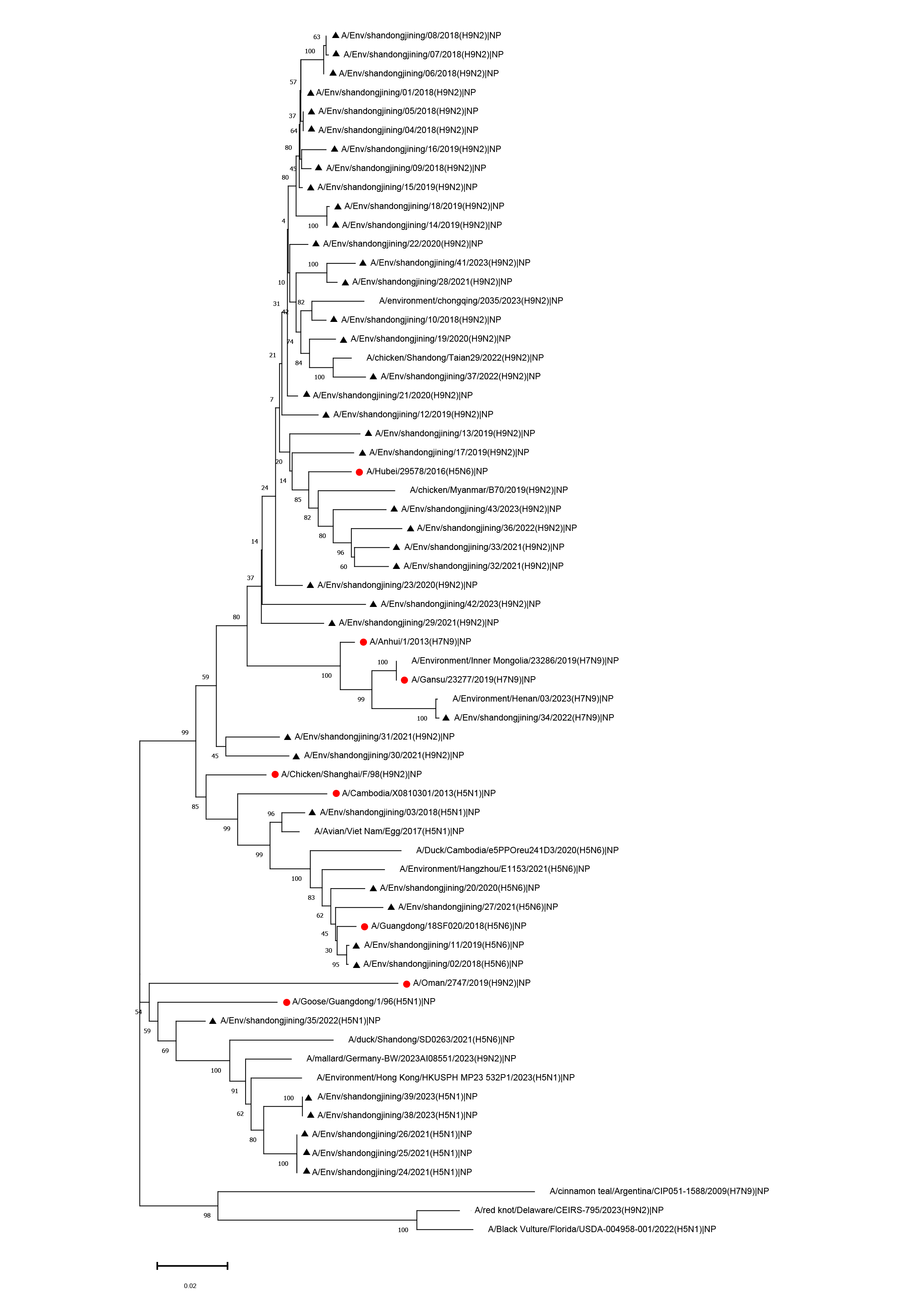

Supplement: SUPPLEMENTARY FIGURE 3 — Phylogenetic tree of NP gene of H5N1, H5N6, H7N9, and H9N2 subtype avian influenza viruses. Phylogenetic analysis of the NP nucleotide sequences of H5N1, H5N6, H7N9 and H9N2 avian influenza viruses in Jining during 2018–2023. “●” represents the vaccine strain. “▲” represents strains sequenced in this study. [file Image_3.TIF]

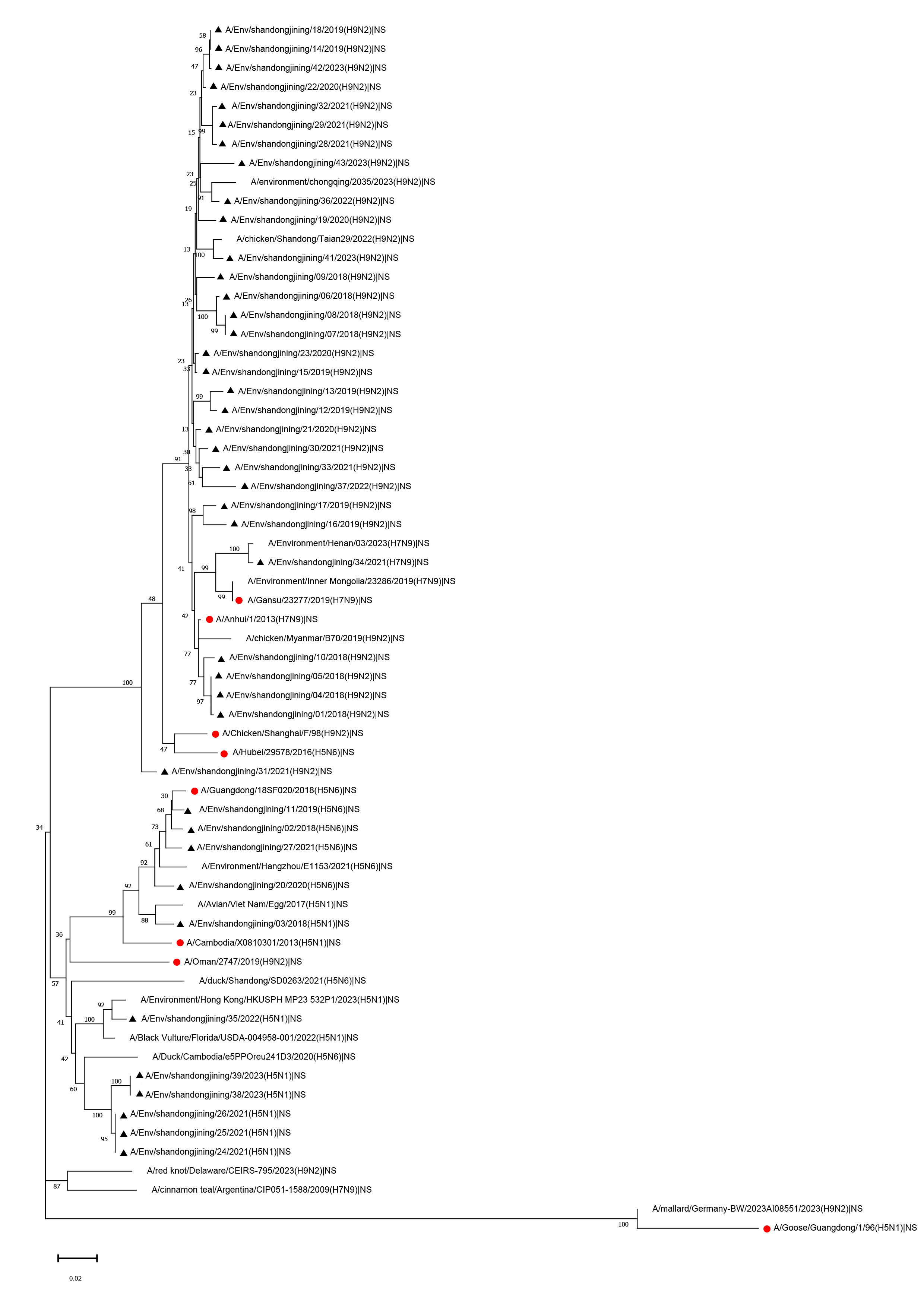

Supplement: SUPPLEMENTARY FIGURE 4 — Phylogenetic tree of NS gene of H5N1, H5N6, H7N9, and H9N2 subtype avian influenza viruses. Phylogenetic analysis of the NS nucleotide sequences of H5N1, H5N6, H7N9 and H9N2 avian influenza viruses in Jining during 2018–2023. “●” represents the vaccine strain. “▲”represents strains sequenced in this study. [file Image_4.TIF]

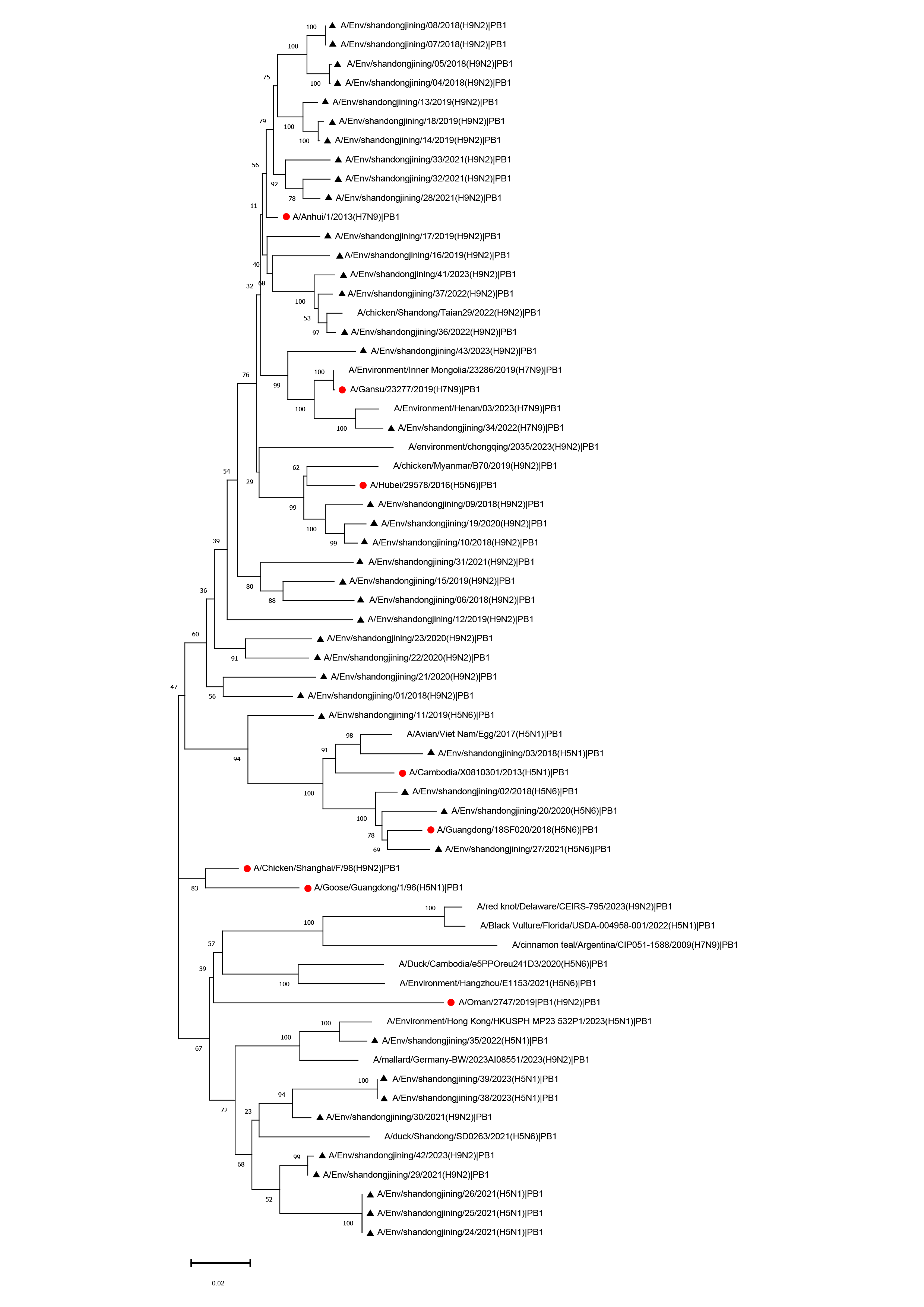

Supplement: SUPPLEMENTARY FIGURE 5 — Phylogenetic tree of PB1 gene of H5N1, H5N6, H7N9, and H9N2 subtype avian influenza viruses. Phylogenetic analysis of the PB1 nucleotide sequences of H5N1, H5N6, H7N9 and H9N2 avian influenza viruses in Jining during 2018–2023. “●” represents the vaccine strain. “▲” represents strains sequenced in this study. [file Image_5.TIF]

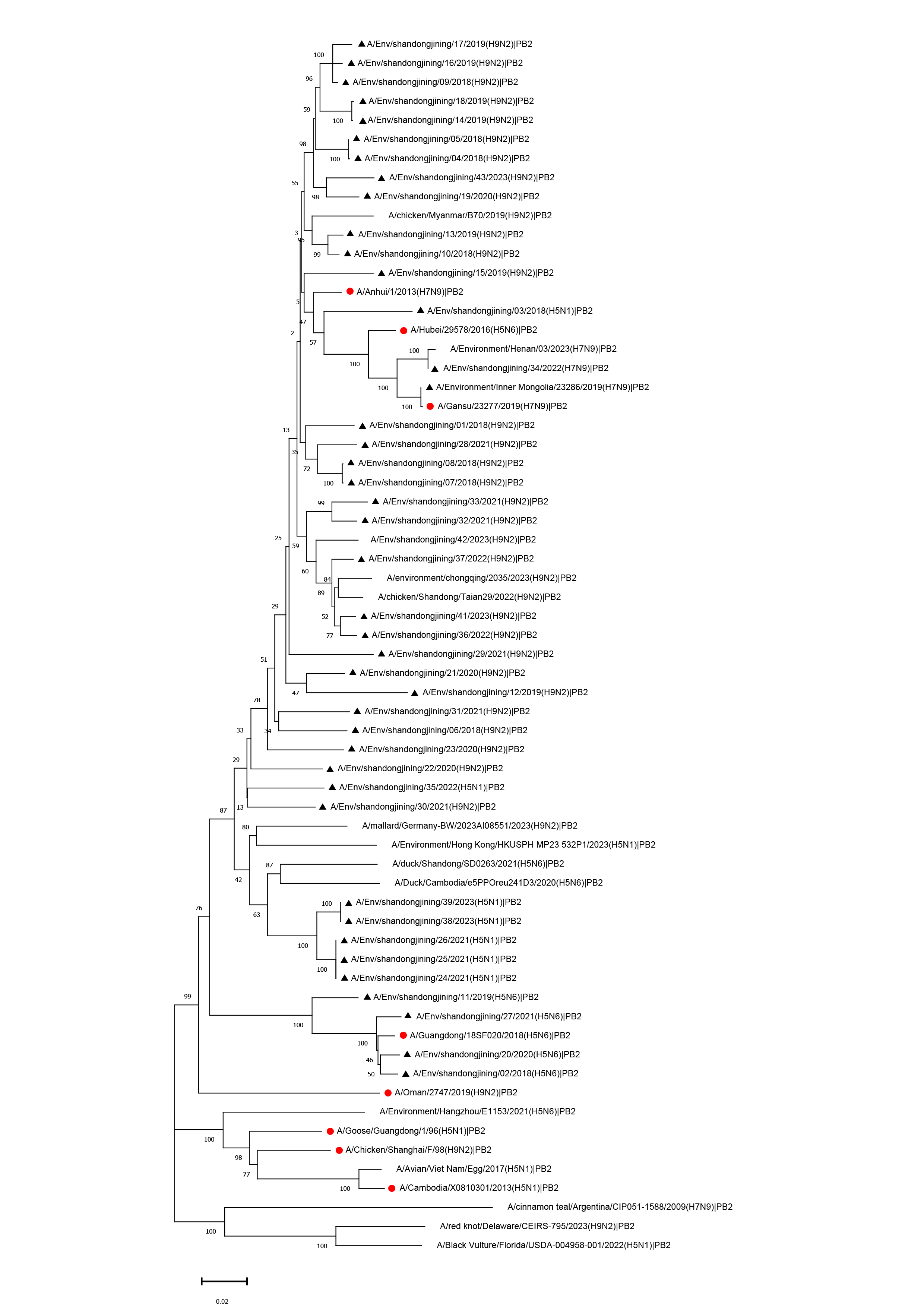

Supplement: SUPPLEMENTARY FIGURE 6 — Phylogenetic tree of PB2 gene of H5N1, H5N6, H7N9, and H9N2 subtype avian influenza viruses. Phylogenetic analysis of the PB2 nucleotide sequences of H5N1, H5N6, H7N9 and H9N2 avian influenza viruses in Jining during 2018–2023. “●” represents the vaccine strain. “▲” represents strains sequenced in this study. [file Image_6.TIF]
