# Supplementary material for: Genetic evolution and molecular characteristics of avian influenza viruses in Jining from 2018 to 2023
Source: Front Microbiol. 2025 Mar 27;16:1551617. doi: 10.3389/fmicb.2025.1551617 (PMC11983498; doi:10.3389/fmicb.2025.1551617)
Supplement: Supplementary file 7 [file Table_1.DOCX]

**Supplement Table 1 Surveillance of avian influenza viruses in Jining from 2018 to 2023.**

| Time | Sample  number | A | | H5 | | H7 | | H9 | | H5/H7 | | H5/H9 | | H7/H9 | | H5/H7/H9 | | HA untyped | |
| --- | --- | --- | --- | --- | --- | --- | --- | --- | --- | --- | --- | --- | --- | --- | --- | --- | --- | --- | --- |
|  |  | Positive number | Positive rate | Positive number | Positive rate | Positive number | Positive rate | Positive number | Positive rate | Positive number | Positive rate | Positive number | Positive rate | Positive number | ­Positive rate | Positive number | Positive rate | Positive number | Positive rate |
| 2018.01-03 | 80 | 16 | 20.00% | 0 | 0.00% | 1 | 1.25% | 8 | 10.00% | 0 | 0.00% | 4 | 5.00% | 0 | 0.00% | 1 | 1.25% | 2 | 2.50% |
| 2018.04-06 | 40 | 5 | 12.50% | 0 | 0.00% | 0 | 0.00% | 5 | 12.50% | 0 | 0.00% | 0 | 0.00% | 0 | 0.00% | 0 | 0.00% | 0 | 0.00% |
| 2018.07-09 | 42 | 5 | 11.90% | 0 | 0.00% | 0 | 0.00% | 5 | 11.90% | 0 | 0.00% | 0 | 0.00% | 0 | 0.00% | 0 | 0.00% | 0 | 0.00% |
| 2018.10-12 | 38 | 5 | 13.16% | 0 | 0.00% | 0 | 0.00% | 5 | 13.16% | 0 | 0.00% | 0 | 0.00% | 0 | 0.00% | 0 | 0.00% | 0 | 0.00% |
| 2019.01-03 | 38 | 3 | 7.89% | 0 | 0.00% | 0 | 0.00% | 0 | 0.00% | 0 | 0.00% | 0 | 0.00% | 0 | 0.00% | 0 | 0.00% | 3 | 7.89% |
| 2019.04-06 | 40 | 3 | 7.50% | 1 | 2.50% | 0 | 0.00% | 1 | 2.50% | 0 | 0.00% | 0 | 0.00% | 0 | 0.00% | 0 | 0.00% | 1 | 2.50% |
| 2019.07-09 | 40 | 7 | 17.50% | 0 | 0.00% | 0 | 0.00% | 7 | 17.50% | 0 | 0.00% | 0 | 0.00% | 0 | 0.00% | 0 | 0.00% | 0 | 0.00% |
| 2019.10-12 | 40 | 6 | 15.00% | 0 | 0.00% | 0 | 0.00% | 6 | 15.00% | 0 | 0.00% | 0 | 0.00% | 0 | 0.00% | 0 | 0.00% | 0 | 0.00% |
| 2020.01-03 | 40 | 0 | 0.00% | 0 | 0.00% | 0 | 0.00% | 0 | 0.00% | 0 | 0.00% | 0 | 0.00% | 0 | 0.00% | 0 | 0.00% | 0 | 0.00% |
| 2020.04-06 | 40 | 2 | 5.00% | 0 | 0.00% | 0 | 0.00% | 2 | 5.00% | 0 | 0.00% | 0 | 0.00% | 0 | 0.00% | 0 | 0.00% | 0 | 0.00% |
| 2020.07-09 | 40 | 7 | 17.50% | 0 | 0.00% | 0 | 0.00% | 7 | 17.50% | 0 | 0.00% | 0 | 0.00% | 0 | 0.00% | 0 | 0.00% | 0 | 0.00% |
| 2020.10-12 | 42 | 13 | 30.95% | 3 | 7.14% | 0 | 0.00% | 7 | 16.67% | 0 | 0.00% | 1 | 2.38% | 0 | 0.00% | 0 | 0.00% | 2 | 4.76% |
| 2021.01-03 | 40 | 10 | 25.00% | 5 | 12.50% | 0 | 0.00% | 4 | 10.00% | 0 | 0.00% | 1 | 2.50% | 0 | 0.00% | 0 | 0.00% | 0 | 0.00% |
| 2021.04-06 | 40 | 6 | 15.00% | 1 | 2.50% | 0 | 0.00% | 5 | 12.50% | 0 | 0.00% | 0 | 0.00% | 0 | 0.00% | 0 | 0.00% | 0 | 0.00% |
| 2021.07-09 | 40 | 8 | 20.00% | 0 | 0.00% | 0 | 0.00% | 6 | 15.00% | 0 | 0.00% | 1 | 2.50% | 1 | 2.50% | 0 | 0.00% | 0 | 0.00% |
| 2021.10-12 | 40 | 0 | 0.00% | 0 | 0.00% | 0 | 0.00% | 0 | 0.00% | 0 | 0.00% | 0 | 0.00% | 0 | 0.00% | 0 | 0.00% | 0 | 0.00% |
| 2022.01-03 | 40 | 10 | 25.00% | 0 | 0.00% | 0 | 0.00% | 10 | 25.00% | 0 | 0.00% | 0 | 0.00% | 0 | 0.00% | 0 | 0.00% | 0 | 0.00% |
| 2022.04-06 | 40 | 5 | 12.50% | 0 | 0.00% | 0 | 0.00% | 3 | 7.50% | 0 | 0.00% | 2 | 5.00% | 0 | 0.00% | 0 | 0.00% | 0 | 0.00% |
| 2022.07-09 | 40 | 3 | 7.50% | 0 | 0.00% | 0 | 0.00% | 2 | 5.00% | 0 | 0.00% | 1 | 2.50% | 0 | 0.00% | 0 | 0.00% | 0 | 0.00% |
| 2022.10-12 | 40 | 11 | 27.50% | 2 | 5.00% | 0 | 0.00% | 5 | 12.50% | 0 | 0.00% | 2 | 5.00% | 2 | 5.00% | 0 | 0.00% | 0 | 0.00% |
| 2023.01-03 | 40 | 12 | 30.00% | 3 | 7.50% | 2 | 5.00% | 6 | 15.00% | 0 | 0.00% | 0 | 0.00% | 1 | 2.50% | 0 | 0.00% | 0 | 0.00% |
| 2023.04-06 | 40 | 10 | 25.00% | 0 | 0.00% | 7 | 17.50% | 0 | 0.00% | 0 | 0.00% | 0 | 0.00% | 0 | 0.00% | 0 | 0.00% | 3 | 7.50% |
| 2023.07-09 | 40 | 2 | 5.00% | 0 | 0.00% | 0 | 0.00% | 2 | 5.00% | 0 | 0.00% | 0 | 0.00% | 0 | 0.00% | 0 | 0.00% | 0 | 0.00% |
| 2023.10-12 | 80 | 11 | 13.75% | 1 | 1.25% | 0 | 0.00% | 9 | 11.25% | 0 | 0.00% | 1 | 1.25% | 0 | 0.00% | 0 | 0.00% | 0 | 0.00% |
| 2018 | 200 | 31 | 15.50% | 0 | 0.00% | 1 | 0.50% | 23 | 11.50% | 0 | 0.00% | 4 | 2.00% | 0 | 0.00% | 1 | 0.50% | 2 | 1.00% |
| 2019 | 158 | 19 | 12.03% | 1 | 0.63% | 0 | 0.00% | 14 | 8.86% | 0 | 0.00% | 0 | 0.00% | 0 | 0.00% | 0 | 0.00% | 4 | 2.53% |
| 2020 | 162 | 22 | 13.58% | 3 | 1.85% | 0 | 0.00% | 16 | 9.88% | 0 | 0.00% | 1 | 0.62% | 0 | 0.00% | 0 | 0.00% | 2 | 1.23% |
| 2021 | 160 | 24 | 15.00% | 6 | 3.75% | 0 | 0.00% | 15 | 9.38% | 0 | 0.00% | 2 | 1.25% | 1 | 0.63% | 0 | 0.00% | 0 | 0.00% |
| 2022 | 160 | 29 | 18.13% | 2 | 1.25% | 0 | 0.00% | 20 | 12.50% | 0 | 0.00% | 5 | 3.13% | 2 | 1.25% | 0 | 0.00% | 0 | 0.00% |
| 2023 | 200 | 35 | 17.50% | 4 | 2.00% | 9 | 4.50% | 17 | 8.50% | 0 | 0.00% | 1 | 0.50% | 1 | 0.50% | 0 | 0.00% | 3 | 1.50% |
| First quarter | 278 | 51 | 18.35% | 8 | 2.88% | 3 | 1.08% | 28 | 10.07% | 0 | 0.00% | 5 | 1.80% | 1 | 0.36% | 1 | 0.36% | 5 | 1.80% |
| Second quarter | 240 | 31 | 12.92% | 2 | 0.83% | 7 | 2.92% | 16 | 6.67% | 0 | 0.00% | 2 | 0.83% | 0 | 0.00% | 0 | 0.00% | 4 | 1.67% |
| Third quarter | 242 | 32 | 13.22% | 0 | 0.00% | 0 | 0.00% | 29 | 11.98% | 0 | 0.00% | 2 | 0.83% | 1 | 0.41% | 0 | 0.00% | 0 | 0.00% |
| Fourth quarter | 280 | 46 | 16.43% | 6 | 2.14% | 0 | 0.00% | 32 | 11.43% | 0 | 0.00% | 4 | 1.43% | 2 | 0.71% | 0 | 0.00% | 2 | 0.71% |
| Total | 1040 | 160 | 15.38% | 16 | 1.54% | 10 | 0.96% | 105 | 10.10% | 0 | 0.00% | 13 | 1.25% | 4 | 0.38% | 1 | 0.10% | 11 | 1.06% |
